# Social isolation, loneliness, and positive affect before and during the COVID-19 pandemic in very old adults living in Germany: a quasi-experimental multi-sample study

**DOI:** 10.1186/s12877-025-06832-6

**Published:** 2025-12-17

**Authors:** Ibrahim Demirer, Jaroslava Zimmermann

**Affiliations:** 1 Department for Psychosomatic Medicine and Psychotherapy, Center for Health Communication and Health Services Research (CHSR), University Hospital Bonn, University Bonn, Venusberg-Campus 1, Gebäude 02/Auenbrugger Haus, Raum 012, Bonn, 53127 Germany; 2 Department of Research Methods, Faculty of Human Sciences, University of Cologne, Cologne, Germany; 3 Cologne Center of Ethics, Rights, Economy, and Social Science of Health, University of Cologne, Cologne, Germany

**Keywords:** Social relationships, Subjective well-being, Oldest old, Quasi-experimental study, Multi-sample, Mediation analysis

## Abstract

**Background:**

Social relationships and the absence of loneliness are vital protective factors for the mental health of ageing individuals. The COVID-19 pandemic poses a dual threat for older adults, exposing them to health risks from SARS-CoV-2 while also restricting access to social resources through public health measures like social distancing. Positive affect, as an ability to experience positive emotions, may help to adapt to stressful situations. However, few studies have explored the impact of the pandemic on the relationship between social resources and positive affect, especially in the very old population, often due to data limitations. This study aims to investigate the cumulative risks of insufficient social resources on positive affect in very old adults before and during the COVID-19 pandemic.

**Methods:**

For a quantitative examination of very old adults (≥ 80 years), we used a pooled multi-sample design incorporating German Ageing Survey (DEAS) waves 3 to 7 and Old Age in Germany (D80+) study. Stratifying the sample into the pre-pandemic (DEAS waves 3–6) and pandemic (DEAS wave 7 and D80+) periods, we identified 8,751 individuals for analysis, with 1,816 measured during and 6,935 individuals before the pandemic. We applied inverse probability of treatment weighting (IPTW) for the likelihood of being part of the DEAS sample and participating during the pandemic. The product of both IPTWs were used in the mediation analysis to assess the association of isolation on positive affect directly and indirectly through loneliness before and during the pandemic.

**Results:**

The highest total effect of social isolation on positive affect occurred during the pandemic (0.283***), with loneliness mediating 34%. Before the pandemic, only the indirect effect of loneliness (-0.097***) remained, with an approximate mediation of 70%. IPTWs effectively balanced and reduced selectivity between the time periods, and results remained robust in sensitivity tests including missing value imputation and error-term correlation.

**Conclusions:**

Cumulative risks during the COVID-19 pandemic escalated, with pandemic responses such as lockdowns increasing the adverse effects of social isolation and loneliness on positive affect of very old adults. Our pooled-multi-sample design allows a quantitative investigation of an understudied population, offering researchers new insights and an empirical strategy to utilize.

**Supplementary Information:**

The online version contains supplementary material available at 10.1186/s12877-025-06832-6.

## Background

The period of the COVID-19 pandemic has been characterized by social distancing in order to minimize the spread of the high-infectious virus - SARS-COV-2. Especially older population has been recommended to stay at home and avoid contact with others due to the increased risk of health complications and mortality in case of infection with SARS-COV-2. There is evidence that older adults have experienced increased social isolation and loneliness during the pandemic [1–4]. Following Coleman [5], social relationships are resources which help to cope with life challenges. More specifically, social relationships may provide information, emotions, or tangibles facilitating an adaption or neuroendocrine reactions to different stressors and in this way they might prevent adverse health responses to stressful events [6]. The COVID-19 pandemic can be viewed as a stressful event, which could doubly penalising older population: firstly, due to the increased risk for serious consequences in case of infection with SARS-COV-2 and secondly because of decreased social resources for coping as a result of the social distancing measures.

A major factor in achieving and maintaining subjective well-being is positive affect. Positive affect refers to the extent to which a person experiences positive emotions such as happiness, joy, and enthusiasm [7] and it has been described as an important indicator of successful aging [8]. In coping and stress research, positive affect is considered a key resource for adapting to stressful situations, such as the COVID-19 pandemic, as it can help broaden the repertoire of coping strategies [7] thereby facilitating more effective stress management [9]. In advanced age, positive affect has been found to be protective for cognitive and mental health [10] or mortality risk [11]. Current studies demonstrated that the COVID-19 pandemic and pandemic-related measures of social distancing have negatively influenced subjective well-being of older adults [12–17]. The evidence confirms that available social resources have protective effect on subjective well-being in older age during the pandemic [12, 15, 18–27]. Research literature often differentiates between objective (quantitative) and subjective (qualitative) aspects of social relationships [28]. Most research studies focusing on the COVID-19 pandemic considered either qualitative, such as perceived social support or feeling of loneliness [18–22, 25], or quantitative aspects of social relationships, such as contact frequency [15, 27, 29, 30]. Few studies considered both of them [12, 24, 26]. Macdonald and Hülür [12] found that higher number of social contacts and higher satisfaction with communication during the pandemic were associated with higher level of positive affect. Moreover, larger social networks and higher frequency of social contacts before and during the pandemic were linked to lower level of loneliness [12]. The findings of Triolo et al. [26] revealed that high pre-pandemic social support was protective against depressive burden during the COVID-19 pandemic, while frequency of social contacts showed no effect. A German representative study [24] reported lower number of social contacts and feeling lonely to be related to increased depression score during the first wave of the COVID-19 pandemic.

In most of the aforementioned studies, the oldest population has been underrepresented [12, 18–24, 26, 29, 30]. Little is known how the COVID-19 pandemic has affected the link between social resources and subjective well-being of the oldest population in Germany as well as in international context. Therefore, the first aim of this study was to examine the process between objective (social isolation) and subjective (feeling of lonely) social resources on positive affect in very old adults living in Germany before and during the COVID-19 pandemic. In addition, there is evidence from previous longitudinal studies showing that social isolation is interrelated with loneliness [31–34]. Considering the COVID-19 pandemic as a specific stressful event, we proposed that lacking of both kind of social resources, objective and subjective, can strengthen the negative effect on subjective well-being. Therefore, the second aim was to test whether a cumulation of social resource shortage contributes to the explanation of variance in positive affect among very old adults examining mediation effect of loneliness on the relationship between social isolation and positive affect.

## Methods

### Study design

For this study we pooled the data from two German population-based representative studies: Old Age in Germany (D80+) [35] and the German Ageing Survey (DEAS) [36]. The studies were conducted independently from each other with different responsible institutes.

### Old age in Germany (D80+)

The study D80+ was funded by the Federal Ministry for Family Affairs, Senior Citizens, Women and Youth (BMFSFJ). The Cologne Centre for Ethics, Rights, Economics, and Social Sciences of Health (ceres) conducted the study with the aim to fill the research gap on individuals aged 80 years or older. The study sample of D80 + is based on all registered persons in Germany living in private households and nursing homes born before March 1st, 1940. The sample was drawn in two stages: (1) population-proportional sampling at community level and (2) disproportionately stratified sampling of population from the community sample (stratified by gender and age). The data collection took place between November 2020 and April 2021, which was the period of the highest infection and death rates in this age group caused by COVID-19 [37]. In total, 40,209 individuals were contacted and 10,360 questionnaires were successfully obtained. Due to the restrictions during the COVID-19 pandemic, a sequential mixed-mode design (i.e., paper-and-pencil survey followed by telephone interview) was used instead of the originally planned face-to-face interviews. A total of 10,578 individuals participated on the survey using a paper-pencil questionnaire (*N* = 10,360) or computer-assisted telephone interviews only (*N* = 218). The most telephone interviews were performed with proxies (*N* = 193) due to the considerable health impairment of target person. A total of 587 participants were living in nursing homes. Persons older than 84 years and men were oversampled in order to enable in-depth subgroup analyses. Additionally, the study set no age-related bounds, leading to an age distribution in the original study sample of 48% between 80-84 years, 31% between 85-89 and 21% who are older than 90 years.

### German ageing survey (DEAS)

The DEAS is conducted by the German Centre of Gerontology and funded by the BMFSFJ. The DEAS started in 1996 and has currently seven waves available (1996, 2002, 2008, 2011, 2014, 2017, 2020/21). The DEAS is a national representative cross-sectional and longitudinal survey (cohort-sequential design) of individuals aged 40 and older who live in private households in Germany. The sampling of the DEAS involves a two-stage sampling procedure (i.e., community, individual). In the first step, 200 communities from West Germany and 90 communities from East Germany were randomly selected (total of 290 communities). The second stage applies disproportionate selection of the target population by sex and age stratified in three age-groups (40–54, 55–69 and 70–85). The DEAS employed this nationally representative sampling strategy for the waves 1996, 2002, 2008 and 2014 (first-time respondents). The current study utilizes all available survey waves of starting DEAS from 2008 (Waves 3–7). The composition of DEAS waves is as follows:

- Wave 3 (2008): Total *N* = 8,218 (panel-respondents *N* = 2,014; first-time respondents *N* = 6,204).
- Wave 4 (2011): Total *N* = 4,980 (panel-respondents)
- Wave 5 (2014): Total *N* = 10,355 (first-time respondents *N* = 6,003; panel-respondents *N* = 4,352)
- Wave 6 (2017): Total *N* = 6,632 (panel-respondents)
- Wave 7 (November 2020 to March 2021): Total *N* = 5,402 (panel-respondents)

Data collection methods varied across the waves: Waves 3 to 6 involved computer-assisted personal interviews and drop-off questionnaires, while Wave 7, due to the COVID-19 restrictions, employed, computer-assisted telephone interviews instead of face-to-face interviews. Additionally, all survey waves were accompanied by a drop-off questionnaire (paper-pencil) containing questions on positive affect, loneliness, and isolation.

### Multi-sample pooling

**Fig. 1:**
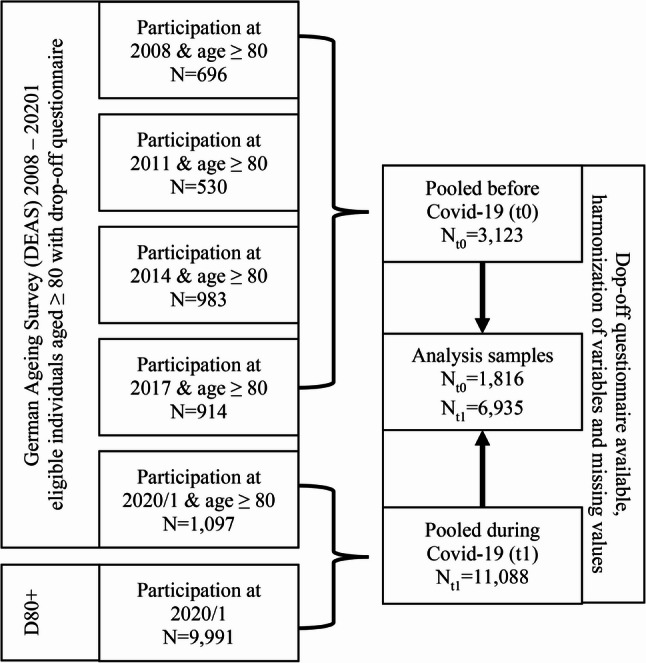
Sample-selection and -pooling process of the DEAS and D80+. Note. Sample restriction criteria: Age ≥ 80; no full inpatient care, participation in drop-off questionnaire in at least one of the waves. N_t0_= before COVID-19 sample consisting of DEAS waves 3–6; N_t1_= during COVID-19 sample consisting of D80+ & DEAS wave 7

Based on the two independent studies of the DEAS and D80+, we wanted to investigate differences in loneliness, isolation and positive affect before and during the COVID-19 pandemic in the oldest old (80 and older). One strategy would have been to analyse the differences based on DEAS waves 6 (2017) and 7 (2020/21) alone. However, this was not reasonable since the amount of observation of individuals aged 80 and older in a single wave of the DEAS is rather low. Therefore, we applied the pooling strategy for the observations before (t0) the COVID-19 pandemic (Wave 3 und to 6 of the DEAS). Similarly, the individuals participated in the D80 + and the DEAS wave 7 were pooled into a during (t1) COVID-19 cross-section. The pooling during COVID-19 (D80+ and DEAS wave 7) was possible, because both studies used the same measurements for the key variables with the same mode of contact and took place between November 2020 and April 2021. For further comparability, the analysis samples excluded individuals within-care facilities from the D80+ (*N* = 587) and only considered variables that were measured identically between the DEAS and D80+. Figure 1 visualizes, this unique data operationalization strategy, that maximized the number of individuals eligible for quantitative analysis while maintaining a temporal structure in the before (t0) and during (t1) COVID-19 time periods.

In total, the pre-pandemic-pool consists of N_t0_=1,816 (pooled data from DEAS wave 3 to 6) individuals with valid entries in all analysis variables, while the during pandemic-pool consists of N_t1_=6,935 individuals (pooled data from D80+ and DEAS wave 7). In combining the DEAS and D80+ datasets, we assumed independence of observations, as the independent sampling frames, the relatively small size of the population aged 80+ relative to the national population, and the geographic spread of the samples make participant overlap highly unlikely.

### Measures

#### Social isolation, loneliness, positive affect

To operationalize objective aspect of social relationships, a binary measure of *social isolation* (no/yes) was used based on Huxbold & Engstler [38]. Participants who were not able to name more than one person in own social network to be in contact with at least once a week were defined as socially isolated. Household members were considered to be a part of the social network. Since this study aims to investigate the interplay between the COVID-19 pandemic social relationships and positive affect, we defined a ‘composite’ or ‘compound’ treatment variable that differentiates between social isolation before and during the COVID-19 pandemic (no/yes) [39]. The aim of such a composite treatment is to categorize the different observable combinations of the treatment (social isolation) and it’s adverse effects given different public health relevant scenarios (COVID-19 pandemic). We define the baseline category with 0, corresponding to the scenario where neither social isolation nor the COVID-19 pandemic took place, assuming the lowest risk of adverse effects on loneliness and positive affect. Individuals who participated in surveys during the COVID-19 pandemic but were not identified as socially isolated were classified as an increased risk group (composite = 1). The second highest risk combination was assigned to survey respondents participating before the COVID-19 pandemic who were identified as socially isolated (composite = 2). Finally, the highest risk group consisted of individuals who participated during the COVID-19 pandemic and were identified as socially isolated (composite = 3).

Subjective aspect of social relationships was represented by feeling of *loneliness*. Loneliness was assessed by one item asking about frequency of feeling lonely in the last week. In both studies, there were four answer options ranging from never to always.

Both studies, DEAS and D80+, used the same version of the PANAS-Scale developed by Watson et al. [40] which measures *positive affect* based on five items with metric values ranging between 1 (never) to 5 (very often). The average value of Cronbach’s alpha was 0.841 in both studies. We used an average score.

#### Confounding variables

Selection on covariates was restricted by the requirement of using identical measures between the studies and the identification of a confounding effect of at least two of the main analysis variables. *Gender* and *age* were identified as main confounding variables. Female gender is associated with having lower subjective well-being [19, 41, 42] and higher levels of loneliness [19, 43]. There is evidence for ageing being associated with decrease in subjective well-being [18, 44] and having fewer social resources [12, 41, 43, 44]. Similarly, we considered *morbidity* as a key confounder, since it can affect the social resources and well-being simultaneously [12, 41, 45]. Morbidity was operationalized as a number of self-reported chronic conditions, which have been medically treated. For *polypharmacy*, we used an information on self-reported number of daily used medications.

Concerning confounding effects on the social resources in more depth, we evaluate the family status as a providing source of social resources. There is evidence that being married have protective effect on loneliness [43, 46] and subjective well-being [20, 42]. Therefore, included a categorized variable of the *family status* with being married (1), single, divorced or separated as one category (2) and widowed (3) as separate categories. Contrastingly, having severe financial restraints, especially in older age, is associated with higher levels of loneliness [43, 46] and lower subjective well-being [20, 21, 41]. We defined having *financial restraints* as being exposed to income poverty (less than 60% of the equalized disposable household in the given survey year).

To capture additional COVID-19 pandemic specific confounding effects, we utilized also information on whether a *SARS-COV-2 infection* has taken place by the respondent her/himself or a person close to her/him close (no/yes) and whether relatives or friends of the respondent *died due to the SARS-COV-2 infection* (no/yes). We organized the confounding variables as a directed acyclic graph (DAGs) and adjusted the main analysis for those variables under the required minimal sufficient adjustment sets (for more details see Appendix Figure S1). Additional variables were also concerned regarding the sample selection processes (see Appendix Equations S1-S3).

### Empirical strategy

Our empirical strategy comprises four steps. Firstly, after the pooling of DEAS and D80 + and discriminating into the pools before and during the COVID-19 pandemic (t0/t1), we aimed to account for non-random systematic differences between the samples and the time points. Although both studies are nationally representative and follow a two-step sampling procedure, DEAS is representative for the individuals aged 40 and older, whereas the D80 + is representative for the individuals aged 80 and older, leading to systematic differences between them. Similarly, individuals that participated during the COVID-19 pandemic (t1) in the survey might be systematically different from those participating before the pandemic (t0).

Therefore, we applied an inverse-probability-treatment weighting (IPTW) strategy for each step of the data selection process. As recommended by Cole & Hernán [47], observational studies can utilize the IPTW strategy to approximate the conditions of a randomized trial by creating a pseudo-population in which the distribution of measured pre-treatment confounders is independent of being in either treatment or control group. This disentanglement from the selection processes is achieved by estimating the probability of receiving the treatment in a numerator and denominator model. The numerator model includes none or only basic covariates (e.g., gender), while the denominator model incorporates more detailed information about the selection process, including potential time-varying confounders. Common diagnostics for the performance of the IPTW include the pseudo-R² statistic of the denominator model and the stability of the IPTW, which should have a mean of around 1 with a reasonable SD (below mean) and no extreme outliers. Unstable IPTW are not desired since they cause certain individuals in the pseudo-population to be weighted too high or low. Importantly, the positivity assumption must be satisfied, meaning all individuals must have a non-zero probability of receiving the treatment. We applied two IPTWs, IPTW-1 aimed to adjust for the selection process that is due to differences in sampling of the DEAS and D80+; IPTW-2 accounted for differences due to different in participation before (t0) or during (t1) the COVID-19 pandemic. The product of both IPTWs was then used to adjust the main analysis, thereby disentangling it from these data selection processes.

Thirdly, the main analysis comprised two marginal structural models (MSM) weighted by the IPTW. MSM 1 estimated the effect of the composite score on loneliness, while MSM 2 estimated the effect of loneliness and the composite score on positive affect. In addition to weighting for the IPTWs, both MSMs, were also adjusted for the confounders (see Appendix Figure S1). Applying IPTWs in the MSMs and then adjusting for additional confounders is superior to conservative regression analysis techniques (e.g., multiple regression) because it considers the bias due to the data generating process (selection bias) process as well as the bias caused by the confounding variables.

Lastly, using the estimates from MSM 1 and MSM 2, we applied the mediational g-formula [48] and calculated the total effect (TE) of social isolation on positive affect and the mediated effects (IE) of loneliness. This approach explicitly shows how different states of the composite score, such as being isolated during the COVID-19 pandemic versus not being isolated before the pandemic, are associated with varying levels of loneliness and positive affect. This allows to differentiate in the association between objective and subjective social resources with positive affect. Detailed formulas of the IPTWs and the mediation are available in the Appendix Equations S1-6.

Finally, we also conducted sensitivity analysis of our results through investigating the success of the balance and success of the IPTWs, and alternate model specifications; the error-term correlation between MSM 1 and MSM 2 and missing value analysis with multiple chained imputation in Appendix Table S2-S3.

## Results

Table 1 contains the descriptive statistics of the t0- and t1- samples after application of the IPTWs (Appendix Equations S1-S3). For comparison, Table S1 in the Appendix contains the unweighted descriptive statistics. Only individuals who were eligible for the analysis - meaning after conducting listwise deletion of missing value – are included. Applying the IPTWs in Table 1, balanced the samples with one another concerning, gender ratio (~ 45%), marital status (~ 47% to 49% married), age (average age of ~ 85), number of daily medication (5 on average) and number of morbidities (3.31 to 3.71).

**Table 1:** Comparison of the weighted descriptive statistics between t0- and t1-sample

	Before COVID-19 pandemic (t0)		During COVID-19 pandemic (t1)	
	Mean/%	SD (Min/Max)	Mean/%	SD (Min/Max)
Positive Affect	3.30	0.52 (1/5)	3.12	0.65 (1/5)
Loneliness	1.30	0.68 (1/4)	1.53	0.70 (1/4)
Confounders				
Age	85.8	3.71 (80/102)	85.2	4.07 (80/102)
Number of morbidities	3.31	1.88 (0/13)	3.5	1.96 (0/13)
Daily medications	5.25	3.6 (0/25)	5.04	3.38 (0/28)
Persons in household	1.67	0.85 (1/8)	1.67	0.73 (1/8)
Female	44.55%		44.91%	
Income poverty	18.72%		16.15%	
Married	47.67%		49.82%	
Divorced/single	8.88%		8.93%	
Widowed	43.44%		41.25%	
East Germany	22.47%		26.59%	
N=	1,816		6,935	
Composite Score (CS)	Not isolated at t0 (CS = 0)	Isolated at t0 (CS = 2)	Not isolated at t1 (CS = 1)	Isolated at t1 (CS = 3)
% in t0 / t1	77.83%	22.17%	89.72%	10.28%
Positive affect	3.32 (0.52)	3.25 (0.54)	3.13 (0.65)	3.06 (0.66)
Loneliness	1.20 (0.57)	1.66 (0.90)	1.51 (0.69)	1.76 (0.78)

Binary indicators contain the percentage in their respective samples instead of the mean values. Standard deviation (SD) omitted for binary indicators. Total *N* = 8,751

In contrast, Table S1 shows clear differences in the pooled-samples: higher age, higher level of loneliness, higher medications use, and higher income poverty in the sample collected during the COVID-19 pandemic. Similarly, positive affect and the percentage of individuals with isolation are lower in the COVID-19 pandemic sample. Furthermore, in the unweighted samples (Table S1) there is a strong selectivity towards lower female rates in the data collected before the COVD-19 pandemic. Table S1 highlights the necessity to address the selectivity between the samples, which have been accounted for by the multiple IPTWs used in Table 1. The first IPTW accounts for the probability of being in one of the DEAS samples (wave 3 to wave 7) (M1) and then the second IPTW for the probability of participating before or during the COVID-19 pandemic (t0/t1) (M2).

Concerning the descriptive distributions of positive affect and loneliness across isolation status exhibited that, on average, individuals during the COVID-19 pandemic had the lowest positive affect (3.06) and the highest loneliness (1.76) scores. In contrast, individuals who were not isolated before the pandemic showed, on average, the highest positive affect (3.32) and the lowest loneliness scores (1.20).

Table 2 contains the model fit statistics for the logistic regression analyses (M1, M2) and the balance of the IPTWs. Table 2 indicates overall good model fits for the prediction of the selectivity. However, M1 showed a higher pseudo r-square with 0.329 than M2 with 0.235. The IPTWs obtained from M1 and M2 have stability with a mean value near one and no extremes in minima or maxima. Likewise, the final IPTW is also stable with a mean value of 1.047 with no outlier weights on any observations. The effectiveness of the IPTWs in reducing selectivity between the samples is also evident in the comparison of Table 1 with Table S1, where the distributions of key variables are balanced after applying the weights.

**Table 2:** Prediction models on sample selectivity

	M1 prob. on D80+	M2 prob. on t1-sample
Mean (SD)	0.711 (0.453)	0.792 (0.406)
Pseudo r-squared	0.329	0.235
Prob > chi2	0.000 (3456.853)	0.000 (2097.558)
Akaike crit. (AIC)	7144.619	6921.858
IPTWs		
Mean (SD)	0.948 (0.709)	0.922 (0.484)
Min/Max	0.290 / 5.410	0.217 / 3.803
	Final IPTW	
Mean (SD)	1.047 (1.436)	
Min/Max	0.063 / 8.677	

Logistic regression used to obtain the probability of positive outcome; M1(1 = D80+), M2(1 = t1-sample). Detailed information on IPTW calculation in Appendix Equation S1-S3

Table 3 presents the mediator model, where the composite score is the predictor on loneliness (MSM 1), and the outcome model (MSM 2), in which both, the composite score and loneliness, are used as predictors on the outcome positive affect (M2). Additionally, Table 3 also contains the mediation parameters, given by the indirect, the total effect and the percentage mediated (%mediated), for each level in the composite score. Detailed formulas for both MSMs and the mediation parameters are available in the Appendix Equations S4-S6. The %mediated expresses the degree of mediation by loneliness at each level of the composite score.

**Table 3:** Results of marginal structural models and mediation parameters

	Marginal structural models		Mediation parameters		
	MSM 1	MSM 2	Indirect effect	Total effect	%Med
Not isolated at t0 (CS = 0) Reference					
Not isolated at t1(CS = 1)	0.295***	-0.151***	-0.055***	-0.206***	26.7%
	[0.200; 0.380]	[-0.223; -0.078]	[-0.068; -0.042]	[-0.313; -0.099]	
Isolated at t0 (CS = 2)	0.413***	-0.033	-0.077**	-0.110	69.9%
	[0.174; 0.652]	[-0.170; 0.104]	[-0.128; -0.025]	[-0.264; 0.044]	
Isolated at t1(CS = 3)	0.523***	-0.186***	-0.097***	-0.283***	34.4%
	[0.401; 0.645]	[-0.286; -0.086]	[-0.134; -0.060]	[-0.362; -0.205]	
Loneliness		-0.186***			
		[-0.221; -0.151]			
R-squared	0.106	0.102			

Confidence intervals (C.I.) in brackets; sig. levels: *** *p* < .001, ** *p* < .01, * *p* < .05. C.I. obtained via bootstrapping with 1.000 replications. Error-term correlation (rho) between MSM 1 & 2: rho = 0.007, rho at which indirect effect is zero: rho=-0.189

In MSM 1, an increase in loneliness is observed with higher levels of the composite score. Specifically, the coefficient for being socially isolated during the COVID-19 pandemic (CS = 3) compared to not being isolated before the pandemic (CS = 0) is significant at 0.523***. Conversely, with decreasing levels in the composite score, the coefficient decreases.

Moving to MSM 2, loneliness exhibits a significantly negative coefficient on positive affect (-0.186***), indicating a decrease in positive affect with increasing levels of in loneliness. Notably, the coefficient for being isolated before the Covid-19 pandemic (CS = 2) is both insignificant and of relatively low magnitude (-0.033), while the coefficients (CS = 1 & CS = 3) during the Covid-19 pandemic are negative and significant. As expected, the magnitudes are highest during the Covid-19 pandemic and being isolated (CS = 3; -0.186***). Both MSMs have a similar goodness of fit and the error-term correlation (rho) between MSM 1 and MSM 2 is very low and insignificant with rho = 0.007, while for the indirect effect to be zero it should have been at least rho=-0.189.

With nearly one half of a standard deviation unit decrease in positive affect the mediation analysis reveals that being isolated during the COVID-19 pandemic (CS = 3) has the highest total effect (TE=-0.283***) and indirect effect (IE=-0.097***). During the COVID-19 pandemic but not being isolated (CS = 1), the total effect is fairly high (TE=-0.206***), while the indirect effect nearly halved (IE=-0.055***) compared to the highest category. The isolated group before the pandemic (CS = 2) shows a more mixed result, with an insignificant and small total effect (TE=-0.110) but a significant and relatively high indirect effect (IE=-0.077***). Consequently, the percentage mediated (%mediated) varies, with the highest at the CS = 2 category (%mediated = 69.9%), driven by the small and insignificant total effect and the relatively high indirect effect. The highest composite score category has the second-highest percentage mediated (%mediated = 34.4%), concurrently holding the highest magnitudes in total and indirect effect. In Appendix Table S3 we also present the same analyses, with imputed data, however, obtaining similar results between the datasets.

## Discussion

Our study examined the association of social resources with positive affect before and during the COVID-19 pandemic in very old individuals living in Germany. We focused on the interplay between social isolation (objective aspect of social relationships) and loneliness (subjective aspect of social relationships) concerning positive affect both before and during the COVID-19 pandemic. Consistent with Coleman [5] and prior research, our findings indicate that insufficient social resources adversely affect the positive affect of very old adults, both before as well as during the COVID-19 pandemic. Notably, before the pandemic, the influence of social isolation on positive affect is entirely mediated by loneliness. However, during the pandemic, the overall strength of the association with social isolation and loneliness on positive affect increased, with social isolation gaining relative importance. These results highlight the relevancy of both objective and subjective aspects of social relationships for very old adults in coping with stressors, such as the period of the COVID-19 pandemic. Accordingly, interventions targeting social resources, such as reducing social isolation, could have been more effective in preventing adverse effects on positive affect during the pandemic.

We analysed data from two nationally representative surveys in Germany, DEAS (waves 3–7) and D80+, focusing on a population that has been relatively understudied quantitatively. Employing a pooled-multi-sample design, our study included 8,751 adults aged 80 and older, with 1,816 individuals surveyed before and 6,935 participants during the COVID-19 pandemic. The pooling of different samples limited our investigation to focus on the measures, which were identical across to the two surveys. Despite the DEAS wave 7 and D80 + employing partially different survey modes during the COVID-19 pandemic, potentially introducing a partial mixed-mode bias, our main analysis focused on variables - namely, isolation, loneliness, and positive affect - that were consistently surveyed via drop-off questionnaires (pen and paper), irrespective of study or survey wave. Fortunately, this consistent approach mitigated potential variations in data collection methods for the core variables of interest. Moreover, concerning the pooling of the during COVID-19 sample, the D80 + and the DEAS wave 7 were both conducted in the same time period during the COVID-19 pandemic, thus reducing unobserved heterogeneity caused by the COVID-19 pandemic.

Regarding potential biases in this study’s findings, three points require closer examination: (1) bias due to missing values, (2) systematic differences between the studies (DEAS vs. D80+), and (3) bias from pooling time points, which may introduce time-varying confounding.

Concerning potential biases of this study’s findings, there are three points that require a closer look, namely, bias through (1) missing values, bias due to (2) systematic differences between the studies (DEAS vs. D80+), bias through pooling (3) the time points and thereby time-varying confounding. The highest relative amount of missing values was found the pooled t0 sample, where the 3,123 individuals aged 80 and older participated but only 1,816 individuals were eligible for final analysis. The highest loss was caused by the fact, that the instrument to measure positive affect is part of the drop-off questionnaire. From these 3,123 individuals 2,301 provided a drop-off questionnaire (~ 73%). The remaining missing values came from the listwise deletion of invalid or missing entries. For these cases, we provided missing value analysis via multiple chained imputations in Table S2 and S3, and found the missing at random assumption to hold.

Although both studies have a similar two-stage sampling procedure and the DEAS aims at representativeness for German adults aged 40 and older, while the D80 + aims at representativeness on the oldest old aged 80 and older, causing systematic differences between the studies (2). We assumed independence of observations, meaning that each respondent contributed only once to the dataset. Although the two studies were independently conducted, the possibility of minimal participant overlap cannot fully be ruled out. However, we observed notable differences in the sample during the COVID-19 pandemic (t1), with a higher participation rate among females and older individuals (see Table S1). Additionally, in the DEAS, there could be stronger self-selection among the oldest old (80 and over), given that the sampling procedure does not attempts to achieve representativeness for this age group in particular. Interestingly, contrary to the existing evidence and our expectations, social isolation was more prevalent in the sample before the COVID-19 pandemic (t0). Such differences hint at different data selection processes that may cause biased estimates. To address these selection processes, we employed multiple IPTWs for each stage of data selection. This is the main advantage of using IPTWs and MSMs over traditional (covariate adjusted multiple regressions) in our study. The use of IPTWs and MSMs aims at disentangling the main analysis from these selection processes. As demonstrated, applying IPTWs for the selection process when conducting a pooled-sample design can greatly reduce differences between the samples, and therefore, we recommend future investigations to make use of this advantage when a traceable data selection process is given. I.

However, despite achieving a generally good fit and stable IPTWs in prediction models M1 and M2 (see also, Table 2; and Appendix Table S2), some level of selectivity persisted. Comparing Table 1 with Table S1 shows that while IPTWs reasonably reduced selectivity, complete elimination did not occur. For example, after applying the IPTWs, the gender ratio shifted from 59/41 to 55/45 in the t0-sample, and the mean age balanced around 85 in t0- and t1-samples, indicating a partial mitigation of selectivity. These remaining differences may hint to remaining time-varying confounding (3). Since we pooled data from 2008 to 2017 in the t0-sample, time-varying differences (e.g., period or cohort effects) are unconsidered. Similarly, the comparison between t0- and t1-sample assumes that these differences are mainly due to the quasi-experimental assignment into the before and during COVID-19 pandemic period, while it is possible that also other events may overlap with this assignment.

Our study confirmed a positive association between social isolation and loneliness, and a negative association of social isolation and loneliness with positive affect. Compared to before the COVID-19 pandemic, the strength of these associations increased. Regarding the mediation analysis further, caution is advised in interpreting the %mediated. Our findings underscore that a higher %mediated may not necessarily correspond to a cumulative risk or total effect, as demonstrated by the isolated group before the COVID-19 pandemic. Despite having the highest %mediated, this group exhibited an insignificant total effect but a significant indirect effect, indicating that loneliness fully mediated the effects of social isolation before the pandemic. This finding is in line with socioemotional selectivity theory [49] demonstrating that social network decreases with increasing age because older adults prioritize to maintain emotionally important social relationships. Thus, the social network size (social isolation) may be less important to older adults than the subjective quality of social relationships (loneliness). Nonetheless, our results provided also an empirical support for the hypothesis of cumulative risks during the COVID-19 pandemic, evident through an increased magnitude of the indirect and total effect. Giving strong support that the COVID-19 pandemic, with associated policies (e.g., social distancing), represented a stressful event which negatively impacted qualitative aspects of social resources and subjective well-being among the very old population in Germany. Very old adults are much more likely to experience health limitations than any other age group and therefore they are more vulnerable to exogenous stressors [50]. Thus, the COVID-19 pandemic might be seen as an additional external stressor that has further restricted resources of the very old, and consequently compromised their ability to adapt to this challenging situation. Therefore, future planning of interventions should also consider their potential impact on this specific vulnerable population group.

Although the pooling strategy into a before and during COVID-19 pandemic has its strengths, a longitudinal investigation with an intra-individual analysis over time could have increased the causal character of this study, and would allow investigation of reciprocities between social resource, positive affect and health in more depths. Such investigation could make use of cross-lagged-panel designs that enable comprehensive tests for reverse-causality issues, which could be especially important regarding social resources and mental well-being [51]. In this context, a recent study by Beller [52] utilized the longitudinal structure of the DEAS, monitoring the mortality association with positive affect and loneliness between 2008 and 2020, highlighting a potential mitigation of the association with mortality through positive affect. However, applying such rigorous panel designs to our studied population was not feasible due to the low quantity of available longitudinal observations in the currently available data. Additionally, biases caused by additional panel attrition/mortality and survivorship bias would severely affect the analysis. Therefore, we opted for a comparison between before (t0) and during (t1) the COVID-19 pandemic as distinct time points. A choice strengthened by the fact that the COVID-19 pandemic acted as a quasi-experiment in survey studies, a situation where respondents had no control over the exogenous event. Still addressing, response selectivity is still a necessity [53] that we aimed through applying the IPTWs for the different stages of data selection. Thus, our study can act as an example for researchers interested in leveraging the quasi-experimental assignment in surveys, to increase the causal interpretability of the empirical strategy.

Regarding the broader implications of social resources and positive affect on outcomes such as mortality [52] and mental health in old age [10], we believe that our findings highlight their significance. Our results indicate an increased association between objective and subjective social resources and positive affect during the COVID-19 pandemic. Given these implications, there is a strong case for policy action on social resources and positive affect, especially during crises like the COVID-19 pandemic.

## Conclusions

Employing a pooled-multi sample design, our study is the first to explore the interrelation between social isolation, loneliness, positive affect in very old adults (80+) living in Germany. Our findings indicate that during the COVID-19 pandemic, both objective (isolation) and subjective social resources (loneliness) demonstrated stronger combined adverse effects on positive affect, whereas before the pandemic, subjective resources primarily contributed to adverse effects on positive affect. Our research design can serve as a guide for future scientists interested in investigating populations with limited available data, such as very old adults.

## Supplementary Information

Supplementary Material 1.

## Data Availability

The data that support the findings of this study are available from German Center of Gerontology (DZA) research data repository. Restrictions apply to the availability of these data. The DEAS (DOI: 10.5156/DEAS.1996-2021.M.002) and D80+ (DOI 10.5156/D80.2022.M.001) are available free of charge to scientific researchers for non-profitable purposes after signing a data distribution contract with the DZA. To request the data please visit: https://www.dza.de/en/research/fdz/access-to-data/application; or contact directly: fdz@dza.deIn addition, questions about the data pooling process can be directed to the corresponding author of this manuscript at ibrahim.demirer@uk-koeln.de.

## Abbreviations

DEAS: German Ageing Survey
TE: Total Effect
IE: Indirect Effect
DE: Direct Effect
IPTW: Inverse-Probability-Treatment-Weight
MSM: Marginal-Structural-Model
CS: Composite Score
%mediated: Percentage mediated
