# Supplementary material for: Social isolation, loneliness, and positive affect before and during the COVID-19 pandemic in very old adults living in Germany: a quasi-experimental multi-sample study
Source: BMC Geriatr. 2025 Dec 17;26:85. doi: 10.1186/s12877-025-06832-6 (PMC12828955; doi:10.1186/s12877-025-06832-6)
Supplement: Supplementary file 1 — Supplementary Material 1. [file 12877_2025_6832_MOESM1_ESM.docx]

Figure S1: Directed Acyclic Graph


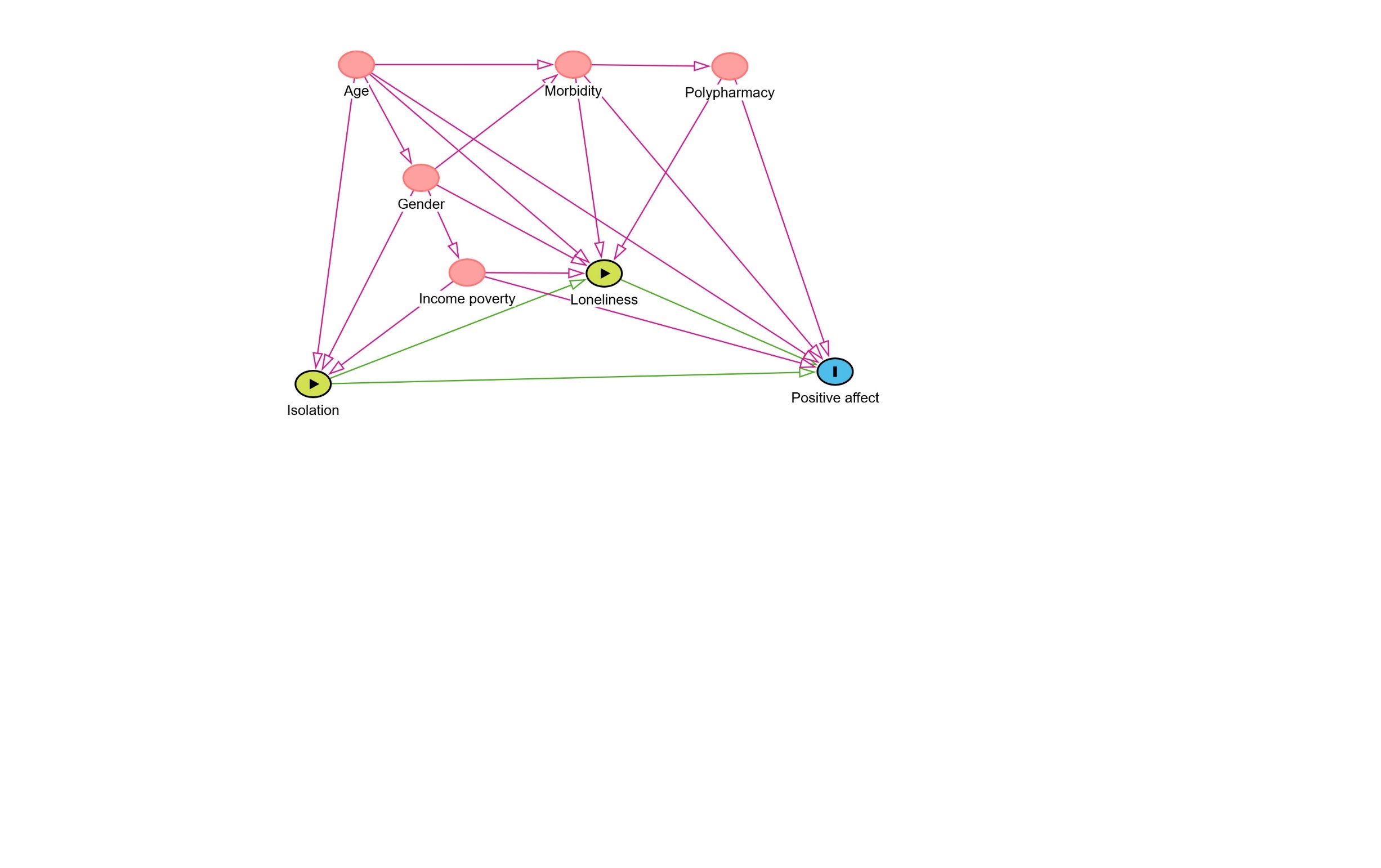


Note. Minimal sufficient adjustment sets for estimating the effect of Isolation, Loneliness (green) on Positive affect (blue): Age, Income poverty, Morbidity, Polypharmacy

Equation S1: sample probability IPTW

$$\hat{P_{S}}=LogReg\hat{P}\left\{ S \right|IncPov, Gender, Age^{2}, Morb, PolyP,FamS, East, HHS\}$$

$$IPTW\left( \hat{P_{S}}=1 \right)=\frac{1-\hat{P}(S)}{\hat{P}(S)};IPTW\left( S=0 \right)=1$$

Equation S2: Covid-19 sample probability IPTW

$$\hat{P_{T}}=LogReg\hat{P}\left\{ T \right|IncPov, Gender, Age^{2}, Morb, PolyP,FamS, East, HHS\}$$

$$IPTW\left( \hat{P_{T}}=1 \right)=\frac{1-\hat{P}(T)}{\hat{P}(T)};IPTW\left( T=0 \right)=1$$

Equation S3: Main analysis IPTW:

$$IPTW\left( \hat{P_{A}} \right)=IPW\left( \hat{P_{T}} \right)* IPTW\left( \hat{P_{S}} \right)$$

Notations: S=1 being part of D80+;T=1 being observed during Covid-1; LogReg = logistic regression; IncPov=Income poverty; Morb=List of morbidities; PolyP=Number of daily Medications; FamS=Family Status; East=Western or Eastern German residence; HHS= Household size.

Equation S4: MSM 1

$$E(M)=\beta_{0}+\beta_{x_{i}}$$

Equation S5: MSM 2

$$E\left( Y \right)=\gamma_{0}+\gamma_{x_{i}}+\gamma_{m}$$

Equation S6: Decomposition of mediation parameters

$$TE=DE+IE=\gamma_{x_{i}}+\left( \beta_{x_{i}*}*\gamma_{m} \right) ; \%mediated=(IE/TE)*100$$

Notations: E(Y) & E(M) are $IPTW\left( \hat{P_{A}} \right)$ weighted generalized linear models using identity link function and cluster robust errors on loneliness (M) or positive affect (Y). Where $\beta_{x_{i}}$ & $\Delta_{x_{i}}$ represent the coefficients of the level ‘i’ in the composite score (x) in the respective MSM. $\Delta_{m}$ the coefficient of loneliness on positive affect. TE= Total Effect, DE= Direct Effect, IE= Indirect Effect.

Table S1: Unweighted descriptive statistics between t0- and t1-sample

|  | Before COVID-19 pandemic (t0) | | During COVID-19 pandemic (t1) | |
| --- | --- | --- | --- | --- |
|  | Mean/% | SD (Min/Max) | Mean/% | SD (Min/Max) |
| Positive Affect | 3.35 | 0.533 (1/4) | 3.11 | 0.676 (1/4) |
| Loneliness | 1.27 | 0.65 (1/5) | 1.57 | 0.712 (1/5) |
| Confounders |  |  |  |  |
| Age | 83.2 | 3 (80/102) | 85.9 | 4.18 (80/102) |
| Number of morbidities | 3.18 | 1.86 (0/13) | 3.41 | 1.95 (0/13) |
| Daily medications | 4.5 | 3.09 (0/25) | 5.17 | 3.31 (0/28) |
| Persons in household | 1.67 | 0.634 (1/8) | 1.69 | 0.795 (1/8) |
| Female | 41.85% |  | 49.82% |  |
| Income poverty | 7.50% |  | 20.06% |  |
| Married | 55.00% |  | 47.90% |  |
| Divorced/single | 8.92% |  | 8.13% |  |
| Widowed | 35.63% |  | 43.97% |  |
| East Germany | 33.60% |  | 22.74% |  |
| N= | 1,816 |  | 6,935 |  |
| Composite Score (CS) | Not isolated at t0 (CS=0) | Isolated at t0 (CS=2) | Not isolated at t1 (CS=1) | Isolated at t1 (CS=3) |
| % in t0 / t1 | 77.83% | 22.17% | 89.26% | 10.74% |
| Positive affect | 3.35 (0.52) | 3.33 (0.57) | 3.11 (0.67) | 3.06 (0.68) |
| Loneliness | 1.20 (0.57) | 1.56 (0.86) | 1.53 (0.57) | 1.80 (0.80) |

Note. Binary indicators contain the percentage in their respective samples instead of the mean values. Standard deviation (SD) omitted for binary indicators. Total N=8,751.

Table S2: Results of multiple chained imputations by Covid-19 sampling (t0/t1)

|  | Before Covid-19 (t0) | | | | During Covid-19 (t1) | | | | Overall | | | | |
| --- | --- | --- | --- | --- | --- | --- | --- | --- | --- | --- | --- | --- | --- |
|  | Complete | Incomplete | Imputed | Total | Complete | Incomplete | Imputed | Total | Complete | Incomplete | Imputed | Total |  |
| Number of Medication | 2833 | 246 | 190 | 3079 | 9878 | 723 | 703 | 10601 | 12711 | 969 | 893 | 13680 |  |
| Income poverty | 2776 | 77 | 77 | 2853 | 8893 | 1645 | 1645 | 10538 | 11669 | 1722 | 1722 | 13391 |  |
| Family (categorical) | 3084 | 4 | 4 | 3088 | 10461 | 141 | 141 | 10602 | 13545 | 145 | 145 | 13690 |  |
| Morbidities: |  |  |  |  |  |  |  |  |  |  |  |  |  |
| CVD | 3093 | 0 | 0 | 3093 | 10103 | 499 | 499 | 10602 | 13196 | 499 | 499 | 13695 |  |
| CHD | 3093 | 0 | 0 | 3093 | 10127 | 475 | 475 | 10602 | 13220 | 475 | 475 | 13695 |  |
| Bloodpressure | 3093 | 0 | 0 | 3093 | 10287 | 315 | 315 | 10602 | 13380 | 315 | 315 | 13695 |  |
| Stroke | 3093 | 0 | 0 | 3093 | 10114 | 488 | 488 | 10602 | 13207 | 488 | 488 | 13695 |  |
| Mental | 3093 | 0 | 0 | 3093 | 10174 | 428 | 428 | 10602 | 13267 | 428 | 428 | 13695 |  |
| Cancer | 3093 | 0 | 0 | 3093 | 10187 | 415 | 415 | 10602 | 13280 | 415 | 415 | 13695 |  |
| Diabetes | 3093 | 0 | 0 | 3093 | 10197 | 405 | 405 | 10602 | 13290 | 405 | 405 | 13695 |  |
| COPD | 3093 | 0 | 0 | 3093 | 10184 | 418 | 418 | 10602 | 13277 | 418 | 418 | 13695 |  |
| Gastrointest. | 2389 | 0 | 0 | 3093 | 9937 | 425 | 425 | 10362 | 12326 | 425 | 425 | 12751 |  |
| Blader / Kidney | 2393 | 0 | 0 | 3093 | 10362 | 0 | 0 | 10362 | 12755 | 0 | 0 | 12755 |  |
| Blood fat | 3093 | 0 | 0 | 3093 | 10125 | 477 | 477 | 10602 | 13218 | 477 | 477 | 13695 |  |
| Arthites | 3093 | 0 | 0 | 3093 | 10249 | 353 | 353 | 10602 | 13342 | 353 | 353 | 13695 |  |
| Urinal | 2395 | 0 | 0 | 3093 | 9947 | 408 | 408 | 10355 | 12342 | 408 | 408 | 12750 |  |
| Glaukoma | 3093 | 0 | 0 | 3093 | 10225 | 377 | 377 | 10602 | 13318 | 377 | 377 | 13695 |  |
| Hearing imp. | 2410 | 0 | 0 | 3093 | 10001 | 349 | 349 | 10350 | 12411 | 349 | 349 | 12760 |  |
| Bloodpress | 3093 | 0 | 0 | 3093 | 10109 | 493 | 493 | 10602 | 13202 | 493 | 493 | 13695 |  |
| Other | 3093 | 0 | 0 | 3093 | 10602 | 0 | 0 | 10602 | 13695 | 0 | 0 | 13695 |  |

Note. Multiple chained imputations with 10 repetitions. Logistic regression on income poverty, and morbidities, multinomial logistic regression on family status, and OLS-regression on number of medications. Using isolation loneliness age and gender as auxiliary predicators. Final N=10,886 with complete information after listwise deletion.

Table S3 MSM 1 MSM 2 and mediation parameters with imputed data

|  | IPTW marginal structural models | | Mediation parameters | | |
| --- | --- | --- | --- | --- | --- |
|  | MSM 1 | MSM 2 | Indirect effect | Total effect | %Med |
| Not isolated at t0 (CS=0) Reference |  |  |  |  |  |
| Not isolated at t1(CS=1) | 0.315*** | -0.131*** | -0.058*** | -0.189*** | 30.7% |
|  | [0.255; 0.376] | [-0.195; -0.067] | [-0.070; -0.046] | [-0.268; -0.110] |  |
| Isolated at t0 (CS=2) | 0.415*** | -0.033 | -0.076** | -0.060 | >100% |
|  | [0.232; 0.598] | [-0.099; 0.123] | [-0.128; -0.025] | [-0.156; 0.036 |  |
| Isolated at t1(CS=3) | 0.489*** | -0.179*** | -0.090*** | -0.269*** | 33.% |
|  | [0.409; 0.569] | [-0.260; -0.098] | [-0.104; -0.076] | [-0.303; -0.235] |  |
| Loneliness |  | -0.184*** |  |  |  |
|  |  | [-0.210; -0.157] |  |  |  |
| R-squared | 0.104 | 0.102 |  |  |  |

*Confidence intervals (C.I.) in brackets; sig. levels: *** p<.001, ** p<.01, * p<.05.* C.I. obtained via bootstrapping with 1.000 replications. Error-term correlation (rho) between MSM 1 & 2: rho=0.008, rho at which indirect effect is zero: rho= -.1833; N=10,886
